# Evidence for exponential progression in advanced Parkinson’s disease: retrospective analysis of a prospective cohort study

**DOI:** 10.3389/fneur.2026.1788633

**Published:** 2026-06-05

**Authors:** Sophie Merve Yener, Catherine Ding, Ganga Ganesvaran, Jane E. Alty, Benjamin G. Clissold, Craig D. McColl, Katrina A. Reardon, Mark Schiff, Peter A. Kempster

**Affiliations:** 1 Neurosciences Department, Monash Medical Centre, Melbourne, VIC, Australia; 2 Tasmanian School of Medicine, University of Tasmania, Hobart, TAS, Australia; 3 Department of Medicine, School of Clinical Sciences, Monash University, Melbourne, VIC, Australia

**Keywords:** cognitive impairment, disease progression, exponential model, motor disability, Parkinson’s disease

## Abstract

**Introduction:**

While prospective cohort research in Parkinson’s disease (PD) suggests a linear increase in motor disability, there is some clinico-pathological evidence for exponential progression in the final disease stage.

**Methods:**

Thirty-four patients with PD composed a long-term cohort. Defined *off* state with levodopa test dose motor scoring on a modified Webster disability scale and Mini-Mental State Examination (MMSE) were conducted every 3 years. Twenty-four patients had 2 or more assessments. For each progression segment, defined as the interval between consecutive assessments, motor progression was calculated as the annual percentage increase of maximum disability score. The entire disease duration is now known for all members of this cohort. Serial motor scores were aligned with time of death, permitting late and earlier observations to be separated for analysis. Progression segments were classified in 3 ways to capture the advanced stage of PD—i) the final segment for each participant; ii) any segment that concluded within 5 years of death; iii) any segment that concluded with a MMSE score < 24. Each advanced category was compared with all other segments from the cohort.

**Results:**

Median age at death was 78.4. Mean disease duration was 14.5 years. Late *off* scores progressed twice as fast, and *on* scores three times as fast, as earlier disease segments (*p* < 0.001 for all comparisons). Classification by presence of cognitive impairment had the strongest effect.

**Discussion:**

Progression of motor disability accelerates in advanced PD. It is possible that the entire disease course has an exponential character.

## Introduction

The assumption that Parkinson’s disease (PD) progresses in a linear manner accords with most clinicians’ impressions and is generally applied in clinical trial design. Published data on longitudinal motor assessments support this view. Virtually without exception, studies with sufficient serial observations to be plotted show linear relationships between motor disability and time (1).

Clinico-pathological research into PD draws scientific power from two sources. The first is pathological ascertainment, though only of the endpoint of a lengthy pathological process. The second, while obvious, is sometimes appreciated less—knowledge of the entire disease course, subject to the amount and quality of clinical documentation. The ability to look backwards can present a different view of progression. One large clinico-pathological study documented the timing of milestones of disease advancement—frequent falling, visual hallucinations, cognitive disability, and need for high level care (2). These milestones had pathological significance. Scoring for Lewy body burden rose in proportion to the number of milestones recorded; multiple clinical milestones corresponded to neocortical Lewy body disease. Alignment of the disease course with time of death showed an unexpected pattern. The length of the advanced PD phase that is heralded by one or more clinical milestones is about the same, at roughly 5 years, irrespective of the duration of the preceding disease course or the age at diagnosis. Patients with young onset PD take decades to reach the advanced disease state. Some patients diagnosed with PD in their eighties develop cognitive decline and other advanced PD milestones within a few years. This observation would not be compatible with a purely linear model of PD progression.

Late non-linear time relationships may be difficult to perceive in prospective PD cohort research, in which most participants do not have advanced PD. Those that do compose only a fraction of the cohort at any point in time. They pass through the advanced phase of the disorder then cease to contribute to follow up data, while survivors continue to dominate the motor scoring statistics.

This paper presents a re-analysis of previously published long term cohort research that conducted motor assessments at regular intervals over more than two decades (3–5). Though composed of a relatively small sample size, the study had a high retention rate and employed rigorous defined *off* state and levodopa test-dose methodology for its motor scoring. Entire disease duration has now been determined for members of the cohort, permitting an alignment of serial motor scores with time of death. This has allowed observations on motor progression in late PD to be separated and compared with progression data for earlier stages.

## Materials and methods

### Participants

The cohort of 34 patients with Parkinson’s disease had been recruited between 1988 and 1995, prior to initiation of levodopa monotherapy. There were 22 men and 12 women; their mean age was 64 years (range 44–87 years). Detailed methodology including entry criteria together with full demographic particulars appear in earlier publications of this study (4, 5).

### Study design and assessments

At 3-year intervals thereafter, a researcher conducted defined *off* state levodopa test dose studies on surviving subjects. Patients’ usual morning levodopa tablets served as the test dose (the mean levodopa dose in the final set of tests was 178 ± 78 mg). This was administered while fasting, all other antiparkinsonian medication having been withheld since the previous evening. The *on* state was defined as the maximal motor improvement 60–90 min post-dose, verified as necessary by repeat scoring. A modified Webster scale (12 areas of motor function scored from 0 to 3 to give a maximum possible motor disability score of 36) was the chief motor assessment (6, 7). The Folstein Mini–Mental State Examination (MMSE) was performed at each visit (8). Two patients dropped out of the study in its early years—one before the first defined *off* state motor assessment and another between the first and second assessment stages. Clinical follow up was otherwise complete and date of death is known for all other participants. Disabilities from advanced PD were strong contributing factors in many cases, but exact cause of death information was not available for the whole cohort and no autopsies were known to have been performed. The study had institutional research ethics approval, and all participants provided written informed consent at each of the 3-yearly stages.

**Figure 1:**
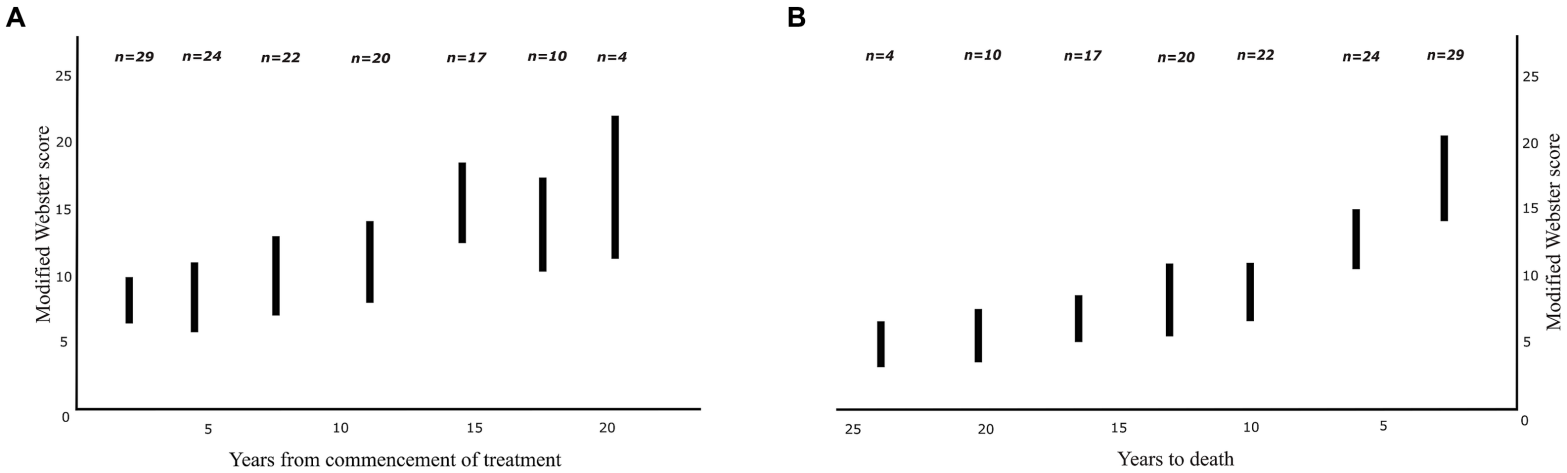
Median *off* (top) and *on* (bottom) modified Webster scores with patient numbers at each 3-yearly assessment. The left panel **(A)** shows measurements prospectively from treatment commencement; the right panel **(B)** shows the same data retrospectively from time of death.

Between 1 and 7 measurements of the levodopa motor response were available from 29 participants who were alive when the first defined *off* state assessments were conducted, and in whom the time between each observation and death is recorded. Figure 1, while not part of the statistical analysis, is included to illustrate the origin of retrospective data. Figure 1A takes the form of previous presentations of this work, showing time of successive assessments from commencement of treatment. In Figure 1B, the assessment stages and numbers of survivors are arranged backwards from time of death.

In 24 participants who survived to attend for two or more assessment stages, rates of progression of *off* and *on* motor scores could be calculated. Progression during a disease segment, defined as the period between consecutive measurements, was the basic unit for comparison. Change in motor score over an observation segment was expressed as a percentage of the maximum motor disability score (36) and divided by the time interval to give percentage *off* and *on* progression per annum (p.a.).

Disease segments were classified in 3 ways to create comparisons between advanced and earlier stages of PD:

1. The final segments for all participants were compared with all earlier segments from the cohort.
2. Segments that concluded within 5 years of death were compared with all other segments from the cohort.
3. Segments that concluded with a MMSE score < 24 were compared with those segments from the cohort that concluded with a MMSE score of 24 or greater (9).

Every o*ff* and *on* measurement was plotted against time to death to examine the curve of best fit for disease trajectory. Linear and exponential curves, as the biologically plausible alternatives, were fitted to these graphs.

### Statistical methods

Statistical analyses were performed with R, version 4.5.0 (R Foundation for Statistical Computing, Vienna, Austria). Normality of continuous variables was assessed using the Shapiro–Wilk test. Descriptive statistics are presented as mean ± standard deviation for parametrically distributed variables and median with interquartile range for non-parametrically distributed variables. Group comparisons were performed using the Mann–Whitney U test. All tests were two-tailed, with statistical significance defined as *p* < 0.05. To visualise progression patterns over time, scatter plots were generated using the ggplot2 package in R. Both linear and exponential regression models were fitted to assess temporal progression, with model performance evaluated using the coefficient of determination (R^2^) and the Akaike Information Criterion (AIC). A lower AIC indicates less information lost when comparing models. With respect to the model with lower AIC, AIC differences (ΔAIC) were classified as: >2, threshold meaningful relative support; >7, moderate relative support; >10, substantial relative support.

## Results

The median age at death for the whole cohort was 78.4 ± 8.8 years. Mean disease duration was 14.5 ± 8.6 years from time of diagnosis, and 14.1 ± 8.0 years from commencement of levodopa treatment. For the 24 final segments, median interval between the segment conclusion and death was 2.6 ± 1.7 years. Across all 97 progression time segments, the interval between segment conclusion and death ranged from 1 month to 25.3 years. The overall median segment duration was 3.2 ± 0.9 years. The median segmental rate of *off* progression was 2.7 ± 4.2% p.a. and the median segmental rate of *on* progression was 1.4 ± 3.3% p.a. Table 1 displays motor progression segments classified by proximity to disease endpoint and presence of cognitive impairment.

**Table 1:** Comparisons of motor progression time segments.

Motor progression time segment comparison (a versus b)	Observation type	a		b		*p*-value
		Median % p.a. (IQR)	n	Median % p.a. (IQR)	n	
Final segments versus all previous segments	*Off*	4.83 (2.93–7.48)	24	2.15 (0.45–4.05)	73	<0.001
	*On*	3.55 (1.52–7.49)	24	0.90 (0.00–2.38)	73	<0.001
Segments ending within 5 years of death versus all other segments	*Off*	4.45 (2.10–6.58)	28	2.17 (0.45–4.07)	69	<0.001
	*On*	3.00 (0.71–6.01)	28	0.93 (0.00–2.38)	69	<0.001
Segments ending with MMSE < 24 versus segments ending with MMSE ≥ 24	*Off*	5.08 (2.27–7.64)	20	2.24 (0.39–4.12)	77	<0.001
	*On*	4.05 (1.72–10.7)	20	0.88 (0.00–2.5)	77	<0.001

IQR, interquartile range; MMSE, Mini Mental State Examination; n, number of segments; p.a., per annum; p values from Mann Whitney U tests.

There are highly significant differences in the rate of motor progression for each of the comparisons in Table 1. For both *off* and *on* measurement, progression was significantly faster (*p* < 0.001) in disease segments that correspond to more advanced PD.

**Figure 2:**
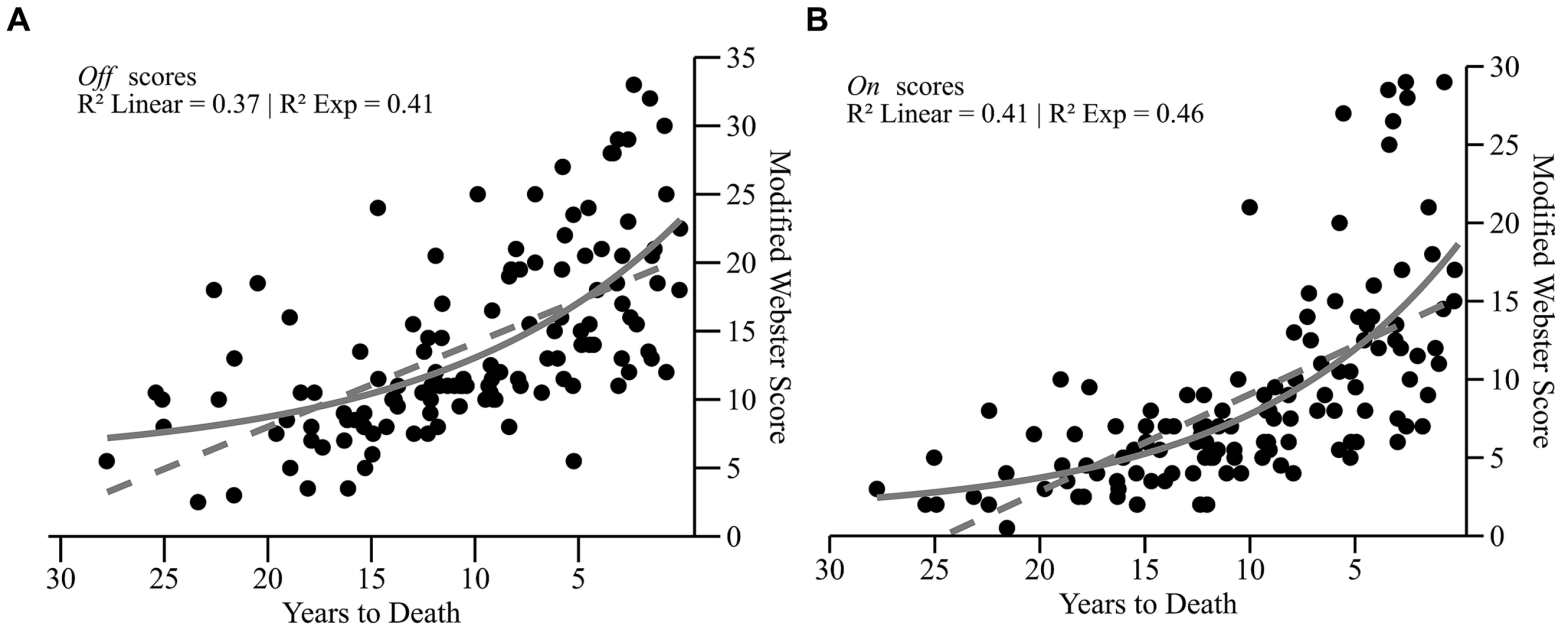
All *off* scores shown in the left panel **(A)** and all *on* scores in the right panel **(B)** plotted against time to death. Linear and exponential curves of best fit are shown.

Figure 2A shows all 126 separate *off* scores and Figure 2B shows all 126 separate *on* scores, plotted against time to death. Linear and exponential curves of best fit have been drawn. Least squares calculation for the exponential curve returns a higher R^2^ statistic than for the linear model (*off* 0.41 versus 0.37; *on* 0.46 versus 0.41). Goodness of fit for these curves was generally better for *on* than for *off*. AIC values were lower for the exponential than linear models (*off* 773 versus 779; *on* 750 versus 760), with ΔAIC values (> 5 for *off* and > 9 for *on*) indicating stronger support for exponential over linear fit.

## Discussion

When the relationship of motor disability scores with the end of the disease course is known, it becomes clear that the rate of progression does accelerate in advanced PD. Whether progression segments are classified by temporal proximity to the disease endpoint or by the presence of the clinical milestone of cognitive impairment, the differences are highly significant. *Off* motor scores progress roughly twice as fast in late compared with earlier PD. The effect is even stronger for *on* scores, which show a three-fold increase. Segment classification based on the MMSE had the greatest influence, where those with low cognitive scores progressed more quickly.

Our findings suggest a non-linear character to the progression of PD. Many biological processes of growth or decay are governed by exponential laws, leading us to compare exponential with linear models of motor progression. But other non-linear mathematical relationships (quadratic polynomial or geometric compounding, for instance) are also possible. In recent years, complex statistical analysis and machine learning methods have been applied to large sets of clinical progression data. This body of research emphasises the heterogeneity of PD progression when variables such as motor phenotype and non-motor disability measures are considered, and suggests that subgroups of patients may follow different trajectories (10–12).

If the conclusions of this retrospective analysis are correct, a neuropathological explanation is likely. Braak had proposed that PD progresses by spread of Lewy pathology across contiguous brain regions (13, 14). It did not take long to realize that this is consistent with a prion-like disease mechanism (15), especially when autopsy studies found Lewy bodies in foetal dopaminergic neurons that had been grafted into the brains of PD patients (16, 17). It is now known that alpha-synuclein aggregates can propagate aggregation of monomers of alpha-synuclein. A disease model based on neuron-to-neuron transmission of alpha-synuclein misfolding would likely involve some exponential behaviour. On the other hand, available evidence does not reveal exponential trends in dopaminergic neuron loss. There have been 4 studies correlating substantia nigra compacta cell counts with PD duration (18–21). Two of these studies proposed a logarithmic pattern of fast then slower degeneration. The other two papers were compatible with linear decline. It is possible that loss of brain network integrity from accumulating neuropathology is contributing to non-linear progression in advanced PD. Network functionality could diminish precipitously once compensatory mechanisms have been degraded by alpha-synuclein deposition (22).

We had previously estimated the rate of progression of *off* scores at 2% p.a. by line of best fit for the staged assessments, and 2.3% by linear regression analysis (3). *On* scores progress somewhat more slowly, which is also shown by other studies using defined *off* and levodopa test-dose methods (1). This aligns with the observation that the levodopa short duration response (the difference between *off* and *on*) is relatively small in early PD, then gradually increases as some patients develop symptomatic motor fluctuations associated with widening margins between *on* and *off* scores (7, 23). Table 1 shows that *on* progression lags well behind *off* progression in measurements conducted during the early and middle disease phases. What is surprising is that the divergence is still present in advanced PD. It is sometimes stated that patients eventually become resistant to levodopa (24, 25). Our findings do not support that idea. Unpredictable variations can reduce the overall effectiveness of levodopa, and the medication delivers less functional benefit at higher levels of motor disability. But it should be possible to reassure patients that a therapeutic response to their dopaminergic drug treatment will continue indefinitely.

Limitations of this research and caveats to its conclusions should be acknowledged. While the study duration is long, the sample of participants is relatively small for PD cohort research. The use of the compact though now obsolete modified Webster scale (maximum score = 36) stems from decisions taken more than 3 decades ago. It is a less sensitive tool than the MDS-UPDRS-III scale (maximum score = 132) (26) or its predecessor, the UPDRS-III (maximum 231 score = 108) (27). Distorted scaling properties at the upper end of PD motor disability scales in general could be a problem. The clinimetric assessment of the MDS-UPDRS was mainly conducted at medium levels of motor disability, but enough scores were obtained to verify scaling performance at its low and high ends (26). There is no evidence from the large body of published research that higher motor disability scores are themselves associated with non-linear patterns on plots of disability versus time, irrespective of motor scale (1). While the Webster scale lacks the clinimetic validation of more modern scoring systems, two studies (including this one) that used it are amongst the longest surveys of serial motor scores (3, 28). Neither showed non-linear characteristics in prospective graphical displays of results. We did not include measurement of medication-induced dyskinesia, which can contribute to progression of disability. Aside from cognitive changes, non-motor symptoms were not captured. There is a further limitation of this retrospective approach to cohort data, which affects clinico-pathological research as well. The assumption that the temporal endpoint of PD corresponds to the final stages of its pathological processes is only approximately correct. Disease-unrelated factors associated with comorbidity and age also influence time of death in chronic neurodegenerative conditions.

It might be argued that the entire PD course is exponential, obscured in early and middle stages by the flatness of the curve and the way that serial observations are usually presented. An exponential model was superior when applied to our entire *on* and *off* datasets, though only at a moderate level of support over the linear model. Nonetheless, an exponential time relationship throughout the disease should be considered when designing clinical trials that depend on outcome measurements of PD progression.

Much larger amounts of PD cohort research data are now available, collected by use of more modern motor assessment tools. But to fully repeat our study, it would be necessary to follow a defined cohort throughout their entire disease course while conducting regular *on* and *off* motor scoring. For this reason, the findings and conclusions reported here are unique at the present time.

## Data Availability

The raw data supporting the conclusions of this article will be made available by the authors, without undue reservation.
